# Giant complete hydatidiform mole: a case report and review of the literature

**DOI:** 10.1186/s13256-024-04474-7

**Published:** 2024-06-13

**Authors:** Iris Bonomo, Suzy Fopa, Grégory Van Vinckenroy, Charlotte Maillard

**Affiliations:** 1 Department of Breast and Reconstructive Surgery, Institut Bergonié, Centre de Lutte Contre le Cancer de Bordeaux, 229 Cours de l’Argonne, 33076 Bordeaux, France; 2 Department of Obstetrics, Cliniques Universitaires Saint-Luc, 1200 Brussels, Belgique; 3 Deparment of Gynecology and Obstetrics, Grand Hôpital de Charleroi, 6000 Charleroi, Belgique; 4 Department of Gynecology and Andrology, Cliniques Universitaires Saint-Luc, 1200 Brussels, Belgique

**Keywords:** Gestational trophoblastic disease, Human chorionic gonadotropin, Hydatidiform mole, Molar pregnancy, Case report

## Abstract

**Background:**

This case describes the youngest patient documented in the literature who presented with a giant hydatidiform mole, effectively addressed through conservative treatment.

**Case presentation:**

Our department received a 20-year-old Caucasian patient who was admitted due to significant metrorrhagia in an undisclosed pregnancy. During examination, we identified a massive, highly vascularized hydatidiform mole measuring 22 cm (cm). We performed a surgical dilatation and curettage. The anatomopathological findings confirmed the presence of a complete hydatidiform mole (CHM). Following the established guidelines, we conducted weekly monitoring of human chorionic gonadotropin (hCG). Unfortunately, the patient discontinued the follow-up and became pregnant again before achieving hCG negativation.

**Conclusion:**

This case suggests that conservative treatment is a viable option regardless of the size of gestational trophoblastic disease (GTD), especially when the preservation of fertility is a crucial consideration, as effectively demonstrated in our case.

## Background

The hydatidiform mole is part of an entity called GTD, and it can manifest as either benign or malignant [1]. These moles arise due to the abnormal development of trophoblastic tissue. The incidence of GTD is influenced by ethnicity, and risk factors include age, family history, parity, personal history of hydatidiform mole, and a history of previous miscarriages [2–4]. The suspicion of the diagnosis is based on an unusually elevated hCG level and abnormalities observed through sonography, with confirmation obtained through histological examination.

Benign lesions can be managed by surgical dilation and curettage [4]. The follow-up involves monitoring hCG level until they turn negative and continuing for an additional 6 months in the case of CHM [4]. Treatment of malignant form relies on systemic treatment, such as chemotherapy.

This case aims to describe the youngest patient ever reported in the literature presenting with a giant hydatidiform mole, successfully managed through conservative treatment.

## Method

This article was written according to the Consensus-based Clinical Case Reporting (CARE) guideline, validated by the Enhancing the QUAlity and Transparency Of health Research (EQUATOR) Network. Ethical approval from the committee was obtained.

## Case presentation

A 20-year-old Caucasian patient was admitted to the emergency due to metrorrhagia. She was gestity 4 and parity 3 with 3 vaginal deliveries in 2017, 2019 and 2020 including one intrauterine fetal demise at 28th weeks without identified cause in 2019. The patient experienced significant vaginal bleeding at home, which had somewhat subsided in intensity upon her arrival at the hospital. She had experienced what she perceived as her period with a menorrhagic tendency the week before admission. The patient was not using any contraception.

Upon her arrival, she presented with a tachycardic at 175 beats per minute and normal blood pressure. The clinical examination indicated a gravid uterus of 22 weeks. Sonography revealed a uterus filled with a heterogeneous, vascularized mass measuring over 15 cm, raising suspicion of molar disease in an unknown pregnancy.

**Fig. 1:**
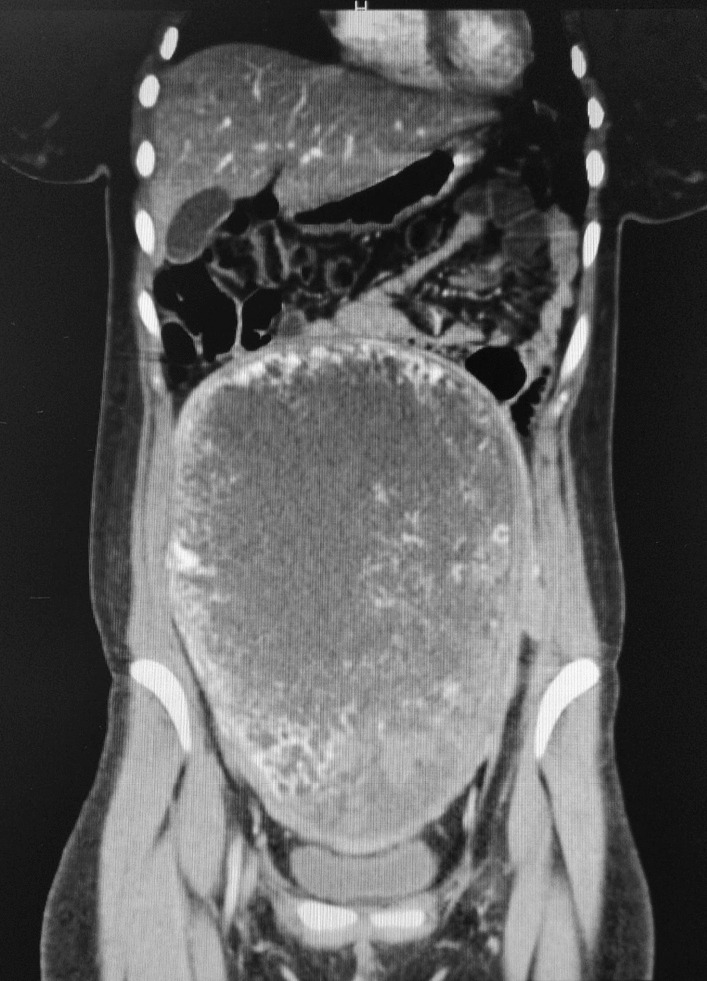
Preoperative thoracoabdominal CT scan

The blood sample indicated an hCG level of 1.193.109 UI/L. The thoracoabdominal computed tomography (CT) scan confirmed a substantial pelvic mass of 22 × 8 × 17 cm, originating from the uterus. The mass appeared mostly heterogenous and liquid, with haemorrhagic zones (Fig. 1). The CT scan did not show distant localization of the disease.

**Fig. 2:**
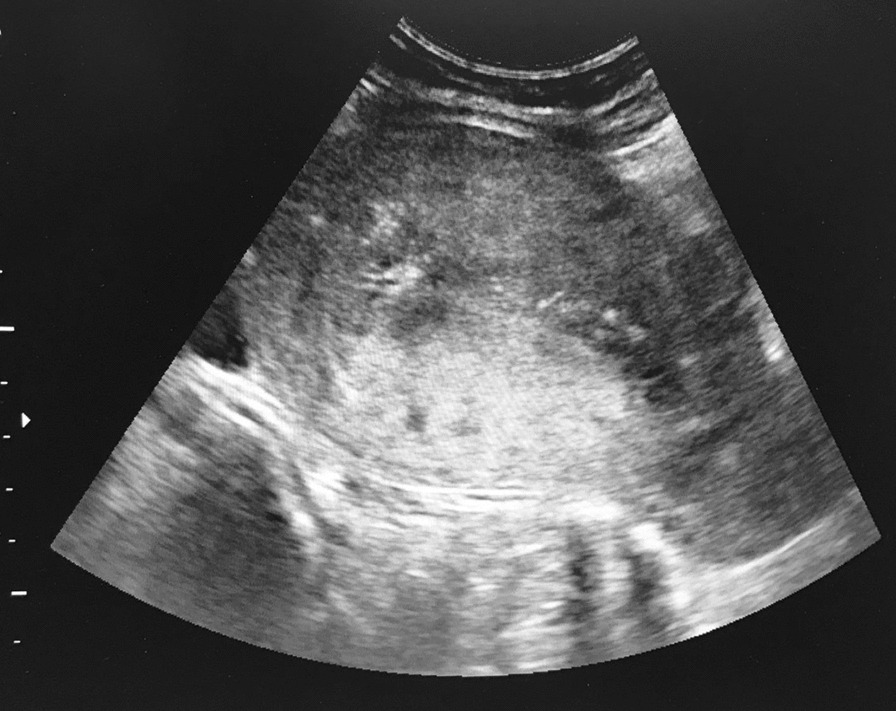
Dilatation and curettage under ultrasound guidance

**Fig. 3:**
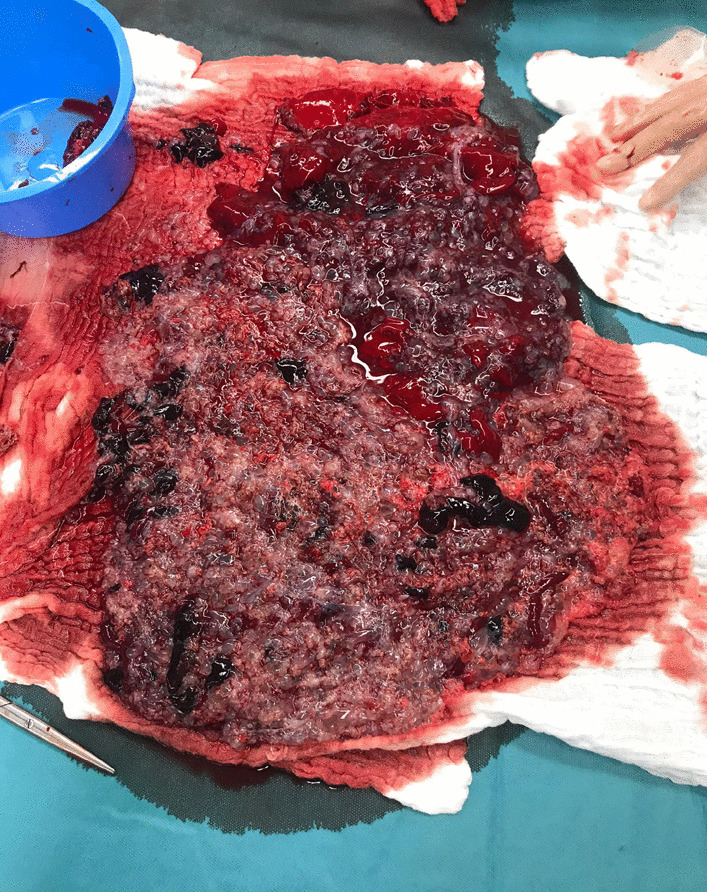
Uterine contents after curettage

Following the advices of the Belgian reference centre of GTD at La Citadelle of Liège, it was recommended to proceed with a surgical dilatation and curettage. Under ultrasound guidance, we evacuated all the uterine contents (Figs. 2, 3). Post-surgery, a transfusion of 3 units of red blood cells was required.

The anatomopathological results showed a CHM weighing 1 kilo with a focus on intra-placental carcinoma. In Belgium, the term “intra-placental carcinoma” is used due to the heightened risk of future choriocarcinoma development in these patients but, it does not change the management. However, this term is not found in the current literature.

A birth control pill was prescribed to the patient. The hCG rate 48 hour after the surgery was 124.000 UI/L. Following established guidelines, we monitored the weekly decrease of the hCG level [5]. Two months after the procedure, the hCG rate was 91 UI/L, representing a decrease over 99.9% of the initial hCG level. Unfortunately, there was a loss of follow-up despite our insistence. Subsequently, we found out that she had been seeking medical attention for a new pregnancy, which had begun around two months after the detection of the CHM. This occurred despite her prescription for contraception and receiving explanations emphasizing the importance of adherence.

## Discussion

GTD are due to a failure of fertilization or gametogenesis. The benign forms include the CHM, partial hydatidiform mole (PHM) and the invasive mole [1]. The malignant forms, called gestational trophoblastic neoplasia (GTN), are the choriocarcinoma (CCA), the placental-site trophoblastic tumor (PSTT) and the epithelioid trophoblastic tumor (ETT).

The incidence of GTD is higher in Asia (1 pregnancy/500) and in Africa/Middle-East (1/1000) than in Europe or North America (1/1500) [2, 6]. Risk factors encompass maternal age (either < 20 years old or > 40 years old), a personal or familial history of molar pregnancy, parity and a history of miscarriage [2–4]. In this particular instance, multiparity, a history of miscarriage, and young age have been identified as risk factors for the development of GTD. The likelihood of a second molar pregnancy following the initial one increases to approximately 1–2%. This risk is significantly higher after a complete mole. The absence of hCG negativation in such cases, along with the detection of a new pregnancy, may potentially obscure the recognition of a GTN. The incidence of GTN is approximately 20% in cases of CHM and 4% in cases of PHM [4].

Histologically, hydatidiform moles are characterized by an excessive proliferation trophoblastic tissue and genetically demonstrate an overexpression of the paternal gene in the placenta [7]. The CHM occurs when an anucleated oocyte is fertilized by one or two sperm, leading to the duplication of its DNA (genotype 46XX or 46 XY). On the other hand, the development of PHM results from a normal egg being fertilized by two sperms (karyotype 69XXY/69XXX/69XYY) [8].

The diagnosis of a hydatidiform mole is suspected through clinical evaluation, ultrasound and the abnormally elevated hCG rate. The accuracy of CHM sonography detection improves with advancing gestational age [9]. At the beginning of the pregnancy, CHM may resemble a blighted ovum or an embryo without cardiac activity. As gestational age progresses, the ultrasound images of CHM adopt the appearance of a “bunch of grapes” or “snowstorm” featuring multiple cystic structures. The uterus is filled with a heterogeneous content with multiple cysts, witnessing the hydropic degeneration of the chorionic villi [1, 10]. As the CHM, the ultrasound detection of the PHM improves with advancing gestational age. The diagnosis is suspected when the placenta is abnormally thick with fetal anomalies [10].

Magnetic resonance imaging (MRI) is recommended for atypical presentation of hydatidiform mole [10]. Pulmonary radiography and CT scan are conducted when dissemination is suspected [11]. In instance of pulmonary metastasis, it is recommended to perform a cerebral MRI.

The standard treatment for hydatidiform mole in patients seeking to preserve their fertility is dilatation and curettage [4]. The anatomopathological examination stays the diagnosis of certainty. A recent meta-analysis conducted by Albright *et al.* [12] shows that the incidence of post-molar GTN in patients who normalize their hCG rate ≥ 56 days after dilatation and curettage is 0.3% in CHM and 0.03% in PHM. Based on this finding, it is rational that the guidelines recommend monthly monitoring of the hCG level after negativation [4, 11]. For PHM, a single negative hCG rate is deemed sufficient [11]. To prevent a new pregnancy during this period, contraception should be introduced.

The treatment of patients suffering from a GTN is determined by the International Federation of Gynecology and Obstetrics (FIGO) score (Table 1) [13]. For cases classified as low-risk cases (0–6), a mono-chemotherapy based on methotrexate at an intramuscular dose of 50 mg. This should be supplemented with folic acid administrated at a 15 mg dose 24 hour after, or alternatively, with Actinomycin D at a dosage of 1.25 mg/m^2^ [14]. For intermediate to high-risk cases (5–6 et > 7), an intravenous polychemotherapy like EMA/CO (Etoposide at 10 mg/m^2^, Methotrexate at 100 mg/m^2^, Actinomycine D at 0.5/m^2^, Cyclophosphamide 600 mg/m^2^ and Vincristine 1 mg/m^2^) is recommended [4, 5, 11, 14]. Although data from 4201 patients including both low and high-risk cases did not indicate any instances of relapse within a 7-year follow-up period, it is still advised to monitor the patient for a duration of 10 years after the normalization of hCG levels [15]. It is possible to consider a new pregnancy 12 months after the hCG rate negativation. For CCA, monitoring of hCG is sufficient. For PSTT/ETT, the follow-up involves an MRI every 6 months for the initial 3 years, followed by annual assessments for a minimum of 5 years [11, 14].

**Table 1:** International Federation of Gynecology and Obstetrics (FIGO) score for gestational trophoblastic neoplasia [12]

Risk factor	0	1	2	4
Age (years)	< 40	≥ 40	/	/
Antecedent pregnancy	Mole	Abortion	term	/
Interval from index pregnancy (months)	< 4	4–< 7	7–12	> 12
Pretreatment serum hCG (iu/l)	< 10^3^	10^3^–10^4^	10^4^–10^5^	> 10^5^
Largest tumor (including uterus)	< 3 cm	3-4 cm	≥ 5 cm	/
Site of metastases	Lung	Spleen, kidney	Gastro-intestinal tract	Brain, liver
Number of metastases	–	1–4	5–8	> 8
Prior failed chemotherapy	/	/	Single drug	≥ 2

Low risk if score of 0–6. Intermediate risk if score of 5–6. High risk if score of > 7

Preserving fertility is crucial when managing cases of PSTT occurring in women of childbearing age. If the uterus is involved, a hysterectomy without salpingo-oophorectomy may be appropriate. In case of myometrial involvement, hysteroscopic resection can be considered Adjuvant chemotherapy should be considered at stages III/IV and, in cases of recurrent risk and persistent hCG, at stages I/II [16, 17].

Seven other cases of giant GTD are found in the literature all occurring in peri- or postmenopausal women [18–24]. They are all found in peri- or postmenopausal women. Except for one case, they were all treated by hysterectomy. In the lone case where suction curettage was performed, complementary chemotherapy by methotrexate was administrated [20]. Out of the seven giant GTD cases described in the literature, only two cases were invasive diseases [18, 23]. This suggests that GTD size should not be considered as a predictive factor for malignancy. Given that our patient was 20-years-old, we aimed to avoid a hysterectomy and performed a curettage. Fortunately, she experienced no complications such as uterine perforation, excessive bleeding or hemoperitoneum. Due to the diagnosis of CHM, chemotherapy was deemed unnecessary.

## Conclusion

To our knowledge, this case represents the youngest patient documented in the literature presenting with a giant hydatidiform mole. It suggests that the size of GTD should not be considered a predictive factor for malignancy. In younger patients, conservative treatment is a feasible option, particularly when preserving fertility is a consideration, as successfully demonstrated in our case.

## Data Availability

The data that support the findings of this case report are available on request from the corresponding author, IB. The data are not publicly available due to information that could compromise privacy.
